# Multi-breed genomic predictions and functional variants for fertility of tropical bulls

**DOI:** 10.1371/journal.pone.0279398

**Published:** 2023-01-26

**Authors:** Laercio R. Porto-Neto, Pamela A. Alexandre, Nicholas J. Hudson, John Bertram, Sean M. McWilliam, Andre W. L. Tan, Marina R. S. Fortes, Michael R. McGowan, Ben J. Hayes, Antonio Reverter

**Affiliations:** 1 CSIRO Agriculture & Food, St Lucia, QLD, Australia; 2 School of Animal Studies, The University of Queensland, Gatton, QLD, Australia; 3 Agriculture Consultant, Livestock Management and Breeding, Toowoomba, QLD, Australia; 4 School of Chemistry and Molecular Bioscience, The University of Queensland, St Lucia, QLD, Australia; 5 School of Veterinary Sciences, The University of Queensland, Gatton, QLD, Australia; 6 Queensland Alliance for Agriculture and Food Innovation, The University of Queensland, St Lucia, QLD, Australia; Universite Clermont Auvergne, FRANCE

## Abstract

Worldwide, most beef breeding herds are naturally mated. As such, the ability to identify and select fertile bulls is critically important for both productivity and genetic improvement. Here, we collected ten fertility-related phenotypes for 6,063 bulls from six tropically adapted breeds. Phenotypes were comprised of four bull conformation traits and six traits directly related to the quality of the bull’s semen. We also generated high-density DNA genotypes for all the animals. In total, 680,758 single nucleotide polymorphism (SNP) genotypes were analyzed. The genomic correlation of the same trait observed in different breeds was positive for scrotal circumference and sheath score on most breed comparisons, but close to zero for the percentage of normal sperm, suggesting a divergent genetic background for this trait. We confirmed the importance of a breed being present in the reference population to the generation of accurate genomic estimated breeding values (GEBV) in an across-breed validation scenario. Average GEBV accuracies varied from 0.19 to 0.44 when the breed was not included in the reference population. The range improved to 0.28 to 0.59 when the breed was in the reference population. Variants associated with the gene HDAC4, six genes from the spermatogenesis-associated (SPATA) family of proteins, and 29 transcription factors were identified as candidate genes. Collectively these results enable very early in-life selection for bull fertility traits, supporting genetic improvement strategies currently taking place within tropical beef production systems. This study also improves our understanding of the molecular basis of male fertility in mammals.

## Introduction

Under a sustainable intensification framework for livestock production, factors that affect the health of humans and animals, and the environment are considered [1]. Bull fertility has such a major impact on the entire beef production system that it is a key phenotype for improvement and an opportunity to assist in meeting the world’s increasing demand for animal protein [2]. Bull fertility is an important economic driver of profitability for beef cattle operations in tropical regions such as northern Australia. Different cattle are better adapted to different environments, resulting in within and between breed variations in fertility traits in response to adverse environmental conditions (e.g. hot humid conditions) [3, 4]. The identification of genetic variants that affect fertility traits, and their application in animal selection would assist in better matching the animal type, production system, and the breeding environment, which can be considered a sustainability goal.

An understanding of male physiology is available [5–7], some relevant genes that affect fertility traits have been identified [8, 9] and genetic parameters have been estimated [10, 11], mainly using pedigree information. However, the implementation of selection approaches for animal improvement, including genomic selection-based programs, have been limited. Compared to beef cow fertility, which has received much consideration and has been managed through the development of reproductive biotechnologies [12, 13] and early stages implementation of genomic-based selection [14, 15], bull fertility lags behind. Currently, in beef cattle, scrotal circumference (SC) is the only bull fertility-related phenotype included in most genetic/genomic evaluation programs. However, during the conduct of a typical bull breeding soundness examination (BBSE) [16] a range of physical, and semen quality traits are objectively assessed. Given the latter has been shown to be heritable [11, 17–19], these traits should be included in male fertility selection programs. This is particularly relevant in tropical environments where there is a risk of high body heat load adversely affecting semen quality.

The multiplicity of breeds used in tropical beef production systems limits the prospect to assemble the large reference populations needed for accurate genomic estimated breeding values (GEBV) for bull fertility. To address this limitation, strategies combining reference populations across breeds using imputed high-density SNP (single nucleotide polymorphisms) genotypes to produce accurate multi-breed GEBV are currently being investigated [15, 20, 21]. Here, we merged six breeds widely used in northern Australia and (sub)tropical regions worldwide to assemble a population of 6,063 bulls measured for ten fertility traits, including four observations on the bull and six observations on the bull’s semen. Using imputed genotypes at high-density (680,758 SNPs), we investigate the accuracy and bias of GEBV using two validation strategies: one where all the phenotypes from one breed are missing from the reference; and the other representing a 5-way cross-validation scheme where a random 20% of phenotypes from all populations are missing from the reference.

This rich dataset was also used to dissect the genomics of bull fertility. Using a 3-way combination of gene network connectivity, pleiotropy, and gene expression in sperm, we identified several candidate functional variants associated with bull fertility traits. Future work could test if these variants aid across breed predictions for bull fertility traits.

## Results and discussion

### Analysis overview

**Fig 1:**
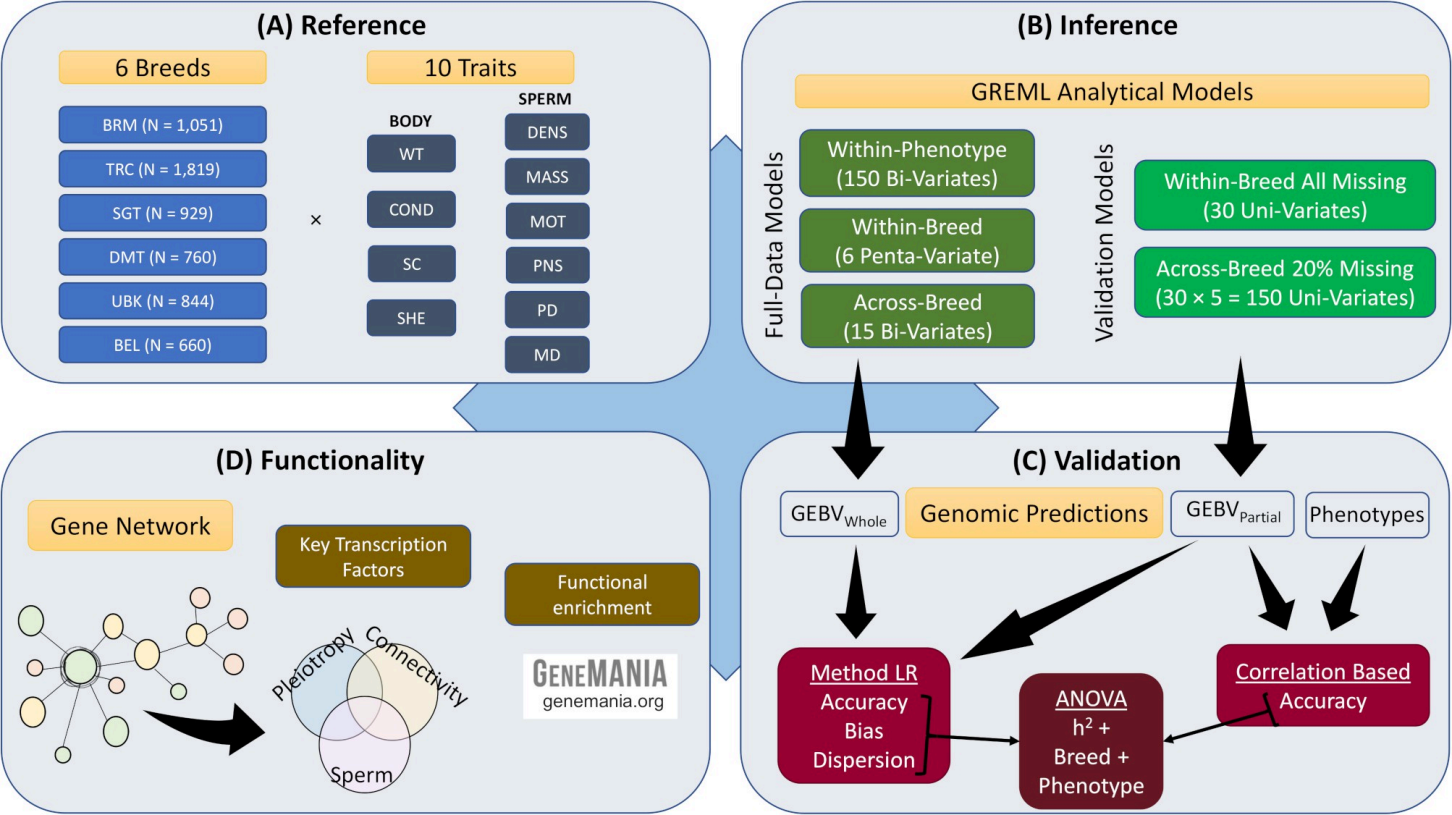
Overview of the analysis. The assembled multi-breed reference population **(A)** comprised 6,063 bulls from six tropically adapted breeds (BRM = Brahman; TRC = Tropical Composite; SGT = Santa Gertrudis; DMT = Droughtmaster; UBK = Ultra Black; and BTC = Belmont Tropical Composite) with measures on 10 fertility-related quantitative phenotypes including four measured on the bull’s body (WT = body weight; COND = condition score; SC = scrotal circumference; and SHEATH = sheath score) and six measured on the bull’s semen (DENS = density; MASS = mass; MOT = motility; PNS = percent normal sperm; PD = proximal droplets; and MD = midpiece deformities). Together with the high-density SNP genotypes, the inference step **(B)** used multi-variate and multi-breed GREML analytical models which can be split into two categories: Full-Data Models with all phenotypic records and used to generate GEBV_Whole_ genomic predictions, and Cross-Validation Models where phenotypes from individuals in the validation group were set as missing values and used to generate GEBV_Partial_ genomic predictions. For the validation step **(C)**, GEBV_Whole_ and GEBV_Partial_ were combined using Method LR approaches to compute accuracy, bias, and dispersion, while GEBV_Partial_ and the adjusted phenotypes were combined to compute correlation-based accuracy. Measures of accuracy, bias and dispersion were subjected to an ANOVA model that contained the effects of heritability (h^2^), breed and phenotype. The final step **(D)** aimed at identifying functional variants. SNP effects were estimated and, when anchored to individual genes, subjected to the Association Weight Matrix to infer a network that exploited the triple concept of degree connectivity, pleiotropy, and sperm expression to identify key transcription factors harbouring causal mutations.

Our approach was to assemble a large reference population of tropically adapted bulls with fertility-related phenotypes and genotyped at high-density to be used to generate multi-breed GEBVs with useful accuracies. A further aim was to explore the factors affecting the accuracy and bias of GEBVs and the identification of functional variants. Our analysis had 4 major steps (detailed description in Materials & Methods):

1. In the Reference step (Fig 1A), a multi-breed population was assembled with 6,063 bulls from six tropically adapted breeds Brahman (BRM), Tropical Composite (TRC), Santa Gertrudis (SGT), Droughtmaster (DMT), Ultra Black (UBK) and Belmont Tropical Composite (BTC) with measures on ten fertility-related phenotypes including measures on the bull Weight (WT, Kg) and body condition score (COND, score) at the same day (or close to) the observation of the other traits, scrotal circumference (SC, cm), sheath score (SHEATH, score) and six measures on the bull’s semen. The semen traits were density (DENS, score), mass activity (MASS, score), percentage of progressive motility (MOT, %), percentage of normal sperm (PNS, %), percentage of sperm cells with proximal cytoplasmic droplets (PD, %) and percentage of sperm cells with middle-piece abnormalities (MD, %). These traits were observed once, primarily collected as routine evaluation and preparation for the bull sale.
2. In the Inference step (Fig 1B), a total of 351 analytical models based on GREML approaches were undertaken to estimate variance components, including heritabilities (h^2^) and genetic correlations, as well as to obtain GEBV. The GREML analyses were performed using a multivariate model using the whole dataset, allowing the estimation of GEBV_Whole_ (or ${\hat{u}}_{w}$ in numerical notation, see Methods) and in uni-variate models for cross-validation after setting to missing values the phenotypes of individuals in the validation population allowing the estimation of GEBV_Partial_ (or ${\hat{u}}_{p}$).
3. In the Validation step (Fig 1C), we compare GEBV_Whole_ and GEBV_Partial_ using Method LR [22] approach to compute the accuracy, bias, and dispersion of GEBVs. Also, we correlate GEBV_Partial_ and the adjusted phenotypes to compute traditional measures of accuracy. Further, using an analysis of variance (ANOVA) model, we explored the strength of the statistical association of h^2^, breed and phenotype on the accuracy, bias, and dispersion of GEBVs.
4. In the Functionality step (Fig 1D), the search for functional variants begun by backsolving the SNP effects from the GREML analyses and subjecting them to the Association Weight Matrix (see Methods) approach to infer a gene co-association network with a focus on regulators shown to have either a high connectivity degree, a significant pleiotropic effect or a high expression value in sperm cells.

### Genetic parameters

The number of records per breed varied from 660 (BTC) to 1,819 (TRC) (S1 Table in S1 File). Descriptive statistics of each trait are presented in S2 Table in S1 File. The traits data were adjusted, before the genetics analyses, for non-genetic effects of population, year of birth, cohort, and the first two principal components (more information on Material and Methods). Multi-breed estimates of genomic heritabilities, genetic and residual correlations are given in Table 1. Using the multi-breed genomic relationship matrix (GRM) (S1 Fig in S1 File) across the 6,063 bulls, 45 bi-variate analyses were performed for as many pair-wise trait combinations using adjusted phenotypes. Each trait was included in nine analyses (one with each of the remaining traits) and the heritabilities listed in Table 1 correspond to the average heritability estimated across the nine analyses.

**Table 1:** Multi-breed estimates of heritability (bold, diagonal), genetic (above diagonal) and residual correlations (below diagonal) from bi-variate analyses.

	WT	COND	SC	SHEATH	DENS	MASS	MOT	PNS	PD	MP
WT	***0*.*37***	0.31	0.43	0.07	0.08	0.08	0.03	-0.08	0.00	0.02
COND	0.34	***0*.*17***	0.11	-0.14	0.07	0.13	0.00	0.09	-0.16	0.05
SC	0.44	0.15	***0*.*47***	0.24	0.17	0.14	0.06	0.05	-0.04	0.05
SHEATH	0.03	0.00	0.04	***0*.*57***	-0.06	-0.07	-0.15	-0.26	0.27	0.12
DENS	0.06	0.00	0.17	0.01	***0*.*20***	0.73	0.34	0.29	-0.18	-0.18
MASS	0.08	0.01	0.15	0.02	0.68	***0*.*21***	0.68	0.52	-0.32	-0.30
MOT	0.05	-0.01	0.11	0.06	0.34	0.63	***0*.*19***	0.53	-0.22	-0.40
PNS	0.11	0.07	0.20	0.05	0.26	0.35	0.35	***0*.*24***	-0.70	-0.51
PD	-0.08	-0.06	-0.17	-0.05	-0.21	-0.22	-0.16	-0.69	***0*.*22***	0.06
MP	-0.04	0.00	-0.08	-0.02	-0.08	-0.16	-0.22	-0.44	0.01	***0*.*22***

A total of 45 bi-variate analyses were performed one for as many pair-wise trait combinations and each using the across-breed GRM. Heritabilities are the average across the 9 bi-variates in which a trait was included.

These reported heritability estimates are well within those published in the literature for the same traits and, on occasions using a subset of this population. For instance, using a population of Brahman (BRM) and Tropical Composite (TRC) bulls, Porto-Neto et al. [23] reported a heritability estimate for sheath score of 0.51 and 0.57 for BRM and TRC, respectively, and very similar to the 0.57 reported here. Similarly, and more recently, for the semen traits, Fortes et al. [19] reported a heritability estimate for PNS (percent normal sperm) of 0.35 and 0.29 for BRM and TRC bulls, slightly higher than the 0.24 found here.

This study expanded previous analyses by including cattle breeds not evaluated before, further validating the genetic relationship between semen traits. The positive correlations (both genetic and residual) between weight, condition score and scrotal circumference have been reported in the past [17], as it was the positive genetic correlations observed for density, mass, motility, and percent normal sperm and the genetically negative correlation between the semen defect traits of PD (proximal cytoplasmic droplets) and MP (midpiece abnormalities) [19]. Importantly, PD and MP were almost uncorrelated (genetic and residual correlation of 0.06 and 0.01, respectively), indicating that these two sperm defects are independent of each other. As such, these genetic parameter estimates offer hope for the possibility of implementing genetic selection programs for these bull fertility traits.

Using bivariate models, we estimated the genomic correlation of the same trait observed in different breeds (Table 2). Except for scrotal circumference (SC) and sheath score, for which the estimated genomic correlations were moderate and positive (i.e., averaged across the 15 breed pairs of 0.36 and 0.51 for SC and sheath, respectively), all other estimates were near zero suggesting a limited contribution of genomic information across breeds. The strong genomic correlations for SC and sheath score might hint at the presence of genomic regions segregating in the populations with a large effect for each trait. Averaged across the 50 bi-variate analyses in which each breed was involved (10 traits × 5 comparing breeds), the estimated genomic correlation was 0.10, 0.13, 0.13, 0.08, 0.09 and 0.09 for BRM, TRC, SGT, DMT, UBK and BTC, respectively. The highest average genomic correlation observed for TRC and SGT suggest GEBVs from these breeds would be more robust in a multi-breed scenario.

**Table 2:** Estimates of genomic correlation within phenotype across all pair wise breeds.

Breed 1	Breed 2	WT	COND	SC	SHEATH	DENS	MASS	MOT	PNS	PD	MP
BRM	TRC	0.73	0.05	0.49	0.67	-0.01	-0.06	-0.04	-0.01	0.00	0.03
BRM	SGT	0.01	0.01	0.62	0.94	0.03	0.01	0.01	0.01	0.00	0.01
BRM	DMT	0.02	0.00	0.43	0.14	0.06	0.01	0.02	0.03	0.03	0.02
BRM	UBK	0.00	0.02	0.37	0.36	0.02	-0.06	-0.08	-0.01	0.01	0.00
BRM	BTC	0.04	0.04	0.03	0.14	0.00	0.00	0.00	0.00	0.00	0.00
TRC	SGT	0.02	-0.03	0.33	0.60	0.10	-0.01	0.02	0.01	0.03	0.06
TRC	DMT	0.01	0.01	0.54	0.02	0.00	-0.08	0.00	-0.01	0.01	0.01
TRC	UBK	0.02	0.14	0.49	0.80	0.08	0.07	0.01	0.03	0.03	0.02
TRC	BTC	0.68	0.07	0.11	0.63	-0.04	-0.04	0.04	0.01	0.00	0.01
SGT	DMT	0.01	-0.16	0.75	0.80	0.00	-0.01	0.00	0.00	0.02	0.01
SGT	UBK	0.01	0.00	0.63	0.57	0.00	0.02	0.00	0.00	0.02	0.00
SGT	BTC	0.01	0.07	0.05	0.94	0.00	0.00	-0.01	0.02	0.01	0.00
DMT	UBK	0.02	0.00	0.28	0.04	0.00	-0.01	-0.01	0.01	0.01	0.01
DMT	BTC	0.02	0.00	0.06	0.68	0.00	0.00	0.00	0.01	0.01	0.00
UBK	BTC	-0.01	0.04	0.27	0.39	0.00	0.00	0.01	0.01	0.00	0.01
Average		0.11	0.11	0.02	0.36	0.51	0.01	-0.01	0.00	0.01	0.01

### Genomic predictions

We did not observe breed differences for the average GEBV within a trait as they were all non-significantly different from zero. However, there were some differences in the GEBV variation across breeds and these differences could reflect different accuracies with higher variations associated with higher accuracies.

**Fig 2:**
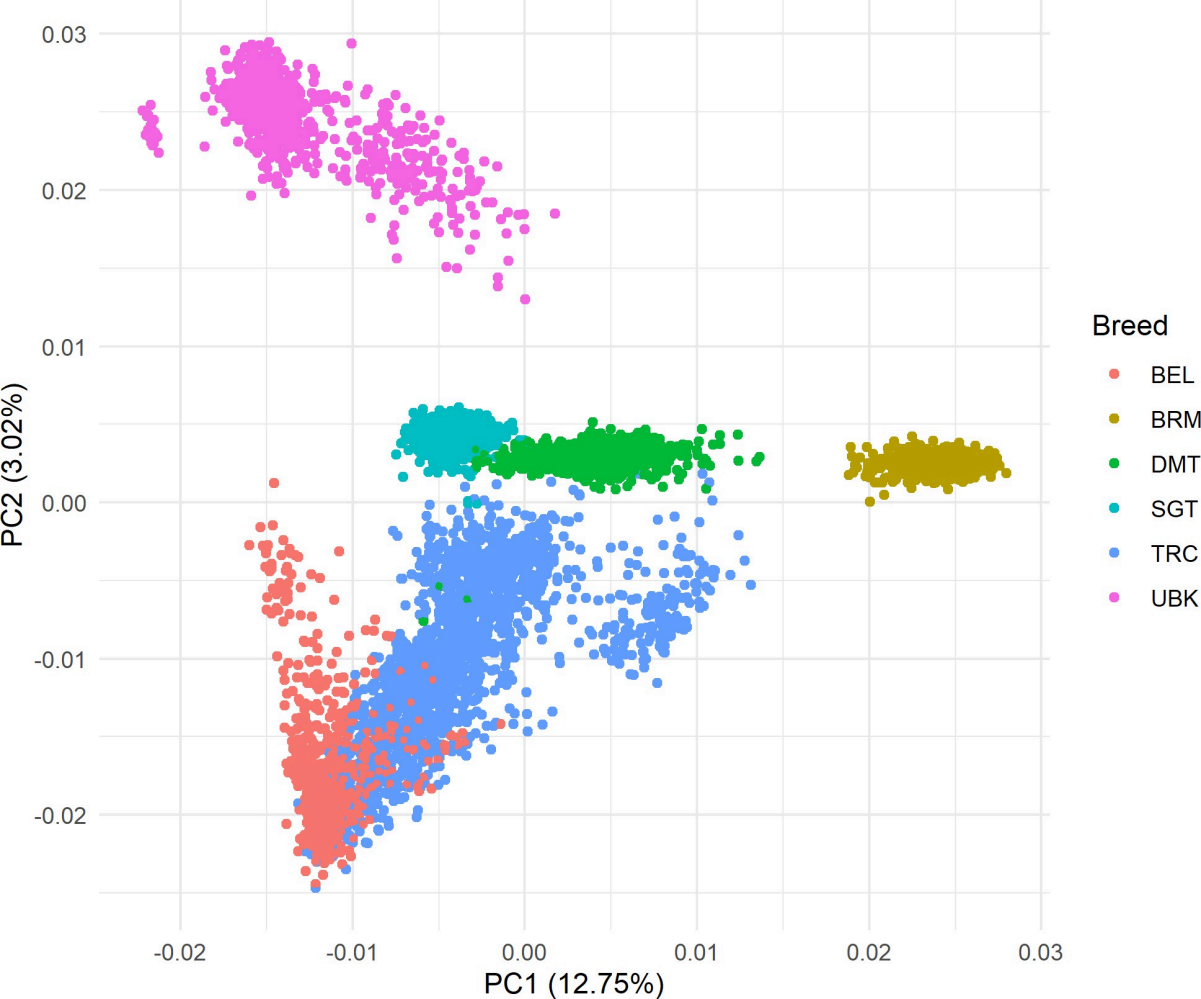
Population structure according to the first two principal components (PC) based on genotypic information of 6,063 bulls. Colors correspond to the six breeds: BTC = Belmont Tropical Composite; BRM = Brahman; DMT = Droughtmaster; SGT = Santa Gertrudis; TRC = Tropical Composite; and UBK = Ultra Black.

Two schemes were used to estimate GEBV accuracies (Table 3): #1) removed a single population completely from the reference and tested the accuracy in predicting its breeding values, and #2) 20% of the observed data were set to missing in five-way cross-validation. GEBV accuracy estimates for a breed, when the breed is not represented in the reference, were lower than those when some animals of the breed are included in the reference (comparison between scheme 1 vs 2), with the largest impact on BRM. This observation was expected, given the known relationship between accuracy and genetic distance to the reference population for a given test animal [24]. Moreover, BRM is the most divergent breed among the six populations (Fig 2), even though it was used during the formation of some of the other breeds.

**Table 3:** Accuracy, bias and dispersion of GEBV across the 10 phenotypes averaged across the 6 breeds and by two validation schemes.

Phenotype	Validation Scheme #1				Validation Scheme #2			
	ACC_R_	ACC_LR_	Bias	Disp.	ACC_R_	ACC_LR_	Bias	Disp.
WT	0.16	0.25	-0.49	0.28	0.44	0.47	0.04	0.03
COND	0.12	0.21	0.00	0.16	0.19	0.32	0.00	0.13
SC	0.36	0.41	-0.03	0.17	0.59	0.59	0.01	0.01
SHEATH	0.44	0.44	0.01	0.09	0.55	0.49	0.00	0.14
DENS	0.03	0.20	0.00	0.25	0.10	0.30	0.00	0.15
MASS	0.00	0.19	0.00	0.27	0.07	0.28	0.00	0.20
MOT	0.06	0.20	0.05	0.24	0.17	0.31	0.01	0.19
PNS	0.11	0.23	0.15	0.30	0.34	0.35	0.01	0.25
PD	0.20	0.24	-0.01	0.15	0.32	0.34	0.01	0.23
MP	0.19	0.23	0.06	0.22	0.29	0.32	0.00	0.30

Validation Scheme #1: From a given validation breed, all measures set as missing in the reference population.

Validation Scheme #2: From a given validation breed, a random 20% of measures are set as missing in the reference (and then averaged across the five 80/20 cross-validation splits).

ACC_R_: correlation-based accuracy. ACC_LR_: method LR accuracy.

To further understand the factors affecting the properties of the multi-breed GEBV reported here, we employed an ANOVA model that contained the effects of heritability estimate, breed, and phenotypic trait as well as the cross-validation sample for the case of the second validation scheme. The results are presented in S3 Table in S1 File. The predictive ability of the model as measured by the coefficient of determination (R^2^) was highest for the accuracy estimated using the method LR (ACC_LR_) than for traditional accuracy (ACC_R_), being 85.1% and 71.9% for validation schemes #1 and #2, respectively. The bias was not affected by any effect included in the ANOVA model indicating the random nature of its variation. Trait and breed were significant sources of variation for both measures of accuracy (S3 Table in S1 File) and dispersion. As in recent attempts at generating multi-breed GEBV [15], genomic linkages can be exploited through the multi-breed GRM. However, the GEBVs produced here are on a different and arbitrary base for each breed. To generate GEBVs on the same base for all breeds, contemporary groups consisting of animals of multiple breeds are required. A promising alternative would be to employ the metafounders approach [25, 26].

### Functional variants

One of the challenges for the implementation of genomic selection strategies in livestock, especially in multi-breed scenarios, is the ability of accurately estimate breeding values across populations and breeds. By focusing more on genomic markers in strong LD with causative variants in combination with denser marker sets or functional subsets of markers, it is possible to leverage information across distantly related breeds to increase the accuracy of genomic predictions [15, 20, 21, 27].

The Association Weight Matrix (AWM) approach exploits patterns of SNP co-association across multiple traits to form connections between the genes harbouring those SNP. The resultant network can be subject to topological analysis that is thought to provide biological insights over and above what can be inferred using traditional approaches such as GWAS. The AWM captured 3,479 SNP-Genes scattered genome-wide, of which 161 genes were annotated as transcription factors (TF). When the AWM was subjected to the PCIT network inference algorithm, 914,955 significant connections between genes were identified. Gene HDAC4 (causal candidate chr3:118,220,595, around 4.8Kbp from the coding gene) was found to have the highest degree (i.e., the higher number of significant connections) with 1,200 connections, making it a key causal candidate. The class II histone deacetylase HDAC4 regulates endocrine functions including male fertility and appetite [28, 29].

With undisputed importance in male fertility, the spermatogenesis-associated (SPATA) family consists of 25 genes (out of 27,607 annotated genes in the bovine genome). Six SPATA genes were captured by the AWM, representing a significant enrichment (P = 0.03, hypergeometric enrichment test). The captured genes were: SPATA16 (chr1:94,583,401), SPATA18 (chr6:67,933,454), SPATA6L (chr8:39,856,393), SPATA2 (chr13:77,990,278), SPATA17 (chr16:21,148,994) and SPATA5 (chr17:34,579,047). Of these, we highlight SPATA6L with 1,139 network connections and its strongest association with the trait PNS, and SPATA16 with 1,058 connections and strongest association with the trait MASS. In fact, a missense variant (p.Ile193Met) in SPATA16 has been recently associated with male fertility of dairy cattle [9]. In addition, the same work identified two coding variants highly associated with bull fertility in ENSBTAG00000019919, another gene captured by our AWM approach that is not fully annotated yet.

The 3-way search for key genes using network connectivity, pleiotropy score and sperm-specific expression, allowed us to prioritise 29 transcription factors (TF) (Table 4, Fig 1) that were ranked in the top 10 positions in at least one of the three criteria. Six of these 29 genes were mapped to chromosome 5 including ARID2, DBX2, YEATS4, HMGA2, SMARCC2, SOX5 and NANOG5. The bovine chromosome 5 has been the subject of great scrutiny. For instance, a literature search on PubMed.gov using the string “bovine chromosome 5” results in 138 publications. Of these, it is worth remarking on the USDA work by McDaneld et al., [30] who identified a deletion on chromosome 5 associated with reproductive efficiency in *Bos indicus*-influenced cattle. However, the association was at around ~10Mbp away from the strongest SNP association here. It is unclear if it relates to the same or a closely located quantitative trait locus. Importantly, and specific for sheath score, Aguiar et al., [31] reported an association of copy number variation at intron 3 of HMGA2 with navel length in *Bos indicus* cattle. In the present study, the SNP in the coding region of HMGA2 (causal candidate: chr5:47,810,529) was found to have the strongest pleiotropic effect of all the SNP-genes in the AWM (chi-square = 327.99; P-value < 10^−16^). The expression of HMGA2 distinguishes between different types of post-pubertal testicular germ cell tumours [32], thus, indicating that HMGA2 has a role in germ cell proliferation, which could affect testicular development in bulls. Moreover, from the 204 genes captured by the AWM for having SNPs associated with sheath score, 104 were located in chromosome 5. Within the network (S2 Fig in S1 File), most genes associated with sheath score create a well-defined cluster, reflecting their cooperative molecular role. Among those, we can highlight HELB (chr5:47,487,358), a gene identified in the context of selection signatures in tropical cattle and proven to segregate independently of the copy number variation HMGA2-CNV [33]. Earlier work also pointed at HELB as a candidate gene for regulating inhibin, produced by Sertoli cells, and shown to be an early biomarker for sexual development in tropical beef cattle [34]. More recently, Xiang et al. [35] reported a cluster of pleiotropic and functional variants on chromosome 5 associated with dairy cattle traits, and Alexandre et al. [36] described a potential indicine-specific peak of chromatin accessibility that agrees with a selective sweep at this genomic region.

**Table 4:** Gene identity and location of candidate causal mutation for 29 TF based on being in the top 10 rank of either of number of network connections, pleiotropy test or expression abundance in sperm.

Gene	Chr	Bp	Connections (Rank)	Pleiotropy (Rank)	Sperm (Rank)
GABPA	1	10,661,504	925 (16)	0.79 (146)	5.07 (10)
BBX	1	52,422,618	151 (140)	2.55 (45)	5.19 (8)
ZNF385B	2	17,312,143	1,038 (3)	0.45 (161)	3.74 (38)
BAZ2B	2	36,738,668	58 (155)	3.85 (19)	5.59 (4)
ATF6	3	7,690,314	730 (38)	4.91 (7)	4.22 (24)
GLIS1	3	92,502,547	43 (160)	4.44 (9)	0.55 (129)
TFEC	4	52,292,757	542 (81)	1.99 (62)	5.39 (6)
ARID2	5	34,527,357	1,023 (4)	1.00 (129)	3.82 (32)
DBX2	5	35,300,281	998 (7)	1.09 (116)	3.74 (37)
YEATS4	5	44,108,845	438 (93)	7.39 (4)	3.63 (42)
HMGA2	5	47,810,529	171 (135)	16.00 (1)	1.99 (88)
SMARCC2	5	57,142,914	169 (136)	4.41 (10)	1.82 (97)
SOX5	5	86,055,295	53 (157)	3.18 (25)	5.66 (3)
NANOG	5	101,425,382	210 (125)	3.46 (24)	5.18 (9)
LCORL	6	37,489,614	458 (91)	7.02 (5)	6.69 (1)
RBPJ	6	45,854,269	998 (8)	1.05 (120)	4.50 (19)
NFIB	8	29,865,913	499 (85)	1.39 (102)	6.16 (2)
ESRRG	16	19,920,181	1,020 (5)	1.54 (92)	4.52 (18)
SMAD1	17	12,828,640	142 (141)	4.68 (8)	3.80 (34)
MZF1	18	65,793,776	1,005 (6)	0.56 (157)	0.00 (161)
NKX2-1	21	46,713,013	1,123 (2)	0.47 (160)	3.50 (45)
MIS18BP1	21	54,958,273	639 (58)	2.34 (50)	5.33 (7)
HMGA1	23	8,315,486	985 (10)	1.67 (80)	1.68 (100)
TFAP2D	23	23,298,001	982 (11)	1.63 (84)	5.41 (5)
ZNF713	25	27,620,537	1,137 (1)	1.05 (122)	3.39 (49)
ZKSCAN1	25	36,398,989	994 (9)	1.49 (95)	0.96 (120)
AFF2	30	31,323,415	514 (83)	7.66 (2)	0.16 (141)
ZNF41	30	85,763,673	91 (146)	7.59 (3)	3.06 (59)
ZNF81	30	86,251,076	176 (132)	6.89 (6)	0.00 (161)

The second cluster of SNP-Genes captured by the AWM was located on chromosome X with 283 genes or 8.13% of the 3,479 in the AWM, compared to 24,058 in the entire panel of 680,758 SNP (or 3.53%, P-value < 0.01). Noting that most genes not shared between species are highly expressed in testis [37], suggested the X chromosome as a promising source of genes associated with spermatogenesis and male fertility. In our AWM, most of the X-mapped genes were associated with PNS, and the ones with the greatest number of connections, highest pleiotropic effect and most expressed in sperm were PLP1 (PNS, chrX:52,547,466), PAK3 (PNS, chrX:59,261,790) and ENSBTAG00000030490 (60S ribosomal protein L17, SHEATH, chrX:102,722,443), respectively. PLP1 was differentially expressed between fresh and frozen-thawed sperm of Holstein bulls [38], with cryodamage being a major problem in semen cryopreservation, causing changes to sperm transcripts that may influence sperm function and motility. Thus, it is tempting to speculate the use of PLP1 as a biomarker of sperm quality or cryotolerance.

**Fig 3:**
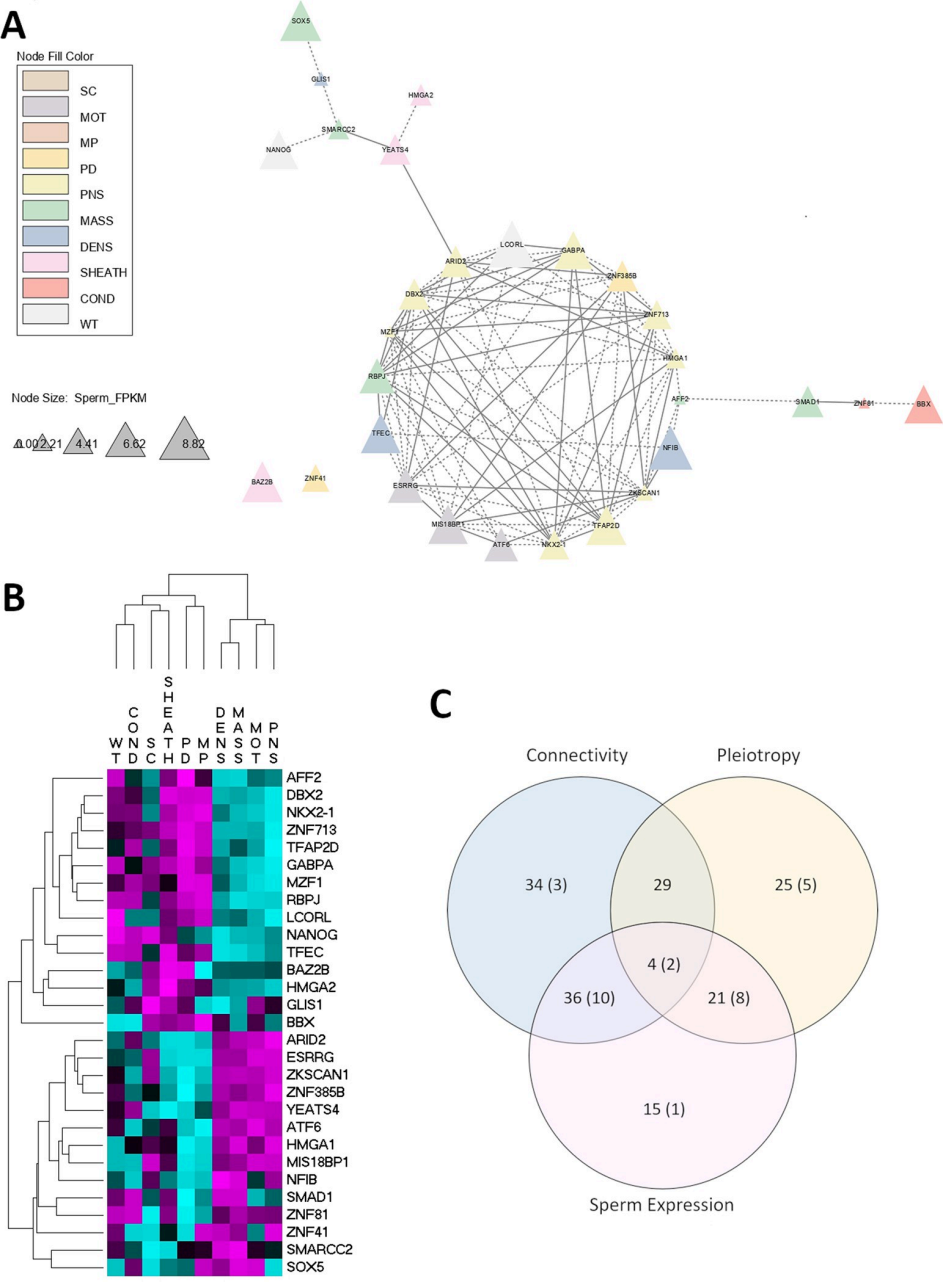
Genes highlighted through functionality analysis. **(A)** Network containing the 29 genes selected as top10 in either connectivity, pleiotropy and/or sperm expression, extracted from a bigger network containing their directly correlated genes (S2 Fig in S1 File)–continuous and dashed edges correspond to positive and negative correlations, respectively; and colors correspond to the most associated trait (SC = scrotal circumference; MOT = motility; MD = midpiece abnormalities; PD = proximal cytoplasmic droplets; PNS = percent normal sperm; MASS = mass; DENS = density; SHEATH = sheath score; COND = condition score; and WT = body weight). **(B)** Co-association scores (AWM output) of the 29 genes. **(C)** Number of transcription factors (TF) selected for being above average according to connectivity, pleiotropy, and sperm expression–the 29 top10 TF for the three selection criteria are represented in parenthesis–Refer to S3 Fig in S1 File, for the complete list of genes.

To further study the 29 transcription factor genes identified by our 3-way search (Fig 3A), we extracted AWM output (co-association scores) for those genes and hierarchically clustered them. Two major trait clusters were identified (Fig 3B): the first one mostly contained the traits measured in the bulls (WT, COND, SC, and SHEATH) plus PD and MP and the second included the other sperm traits (DENS, MASS, MOT and PNS). This separation between sperm traits is probably because for PD and MP the smaller the value the better, while the opposite is true for DENS, MASS, MOT and PNS. In terms of the genes, also two clusters can be observed potentially indicating different biological functions.

Finally, when we ran a functional enrichment analysis using the 29 key TF (S4 Table in S1 File), apart from finding expected enriched terms related to DNA binding, transcription, and chromatin remodelling, we also found “stem cell differentiation” (FDR = 4.51E-03) and “regulation of stem cell differentiation (FDR = 2.39E-02). The enrichment was due to five genes, namely NANOG (chr5: 101,425,382), HMGA2 (chr5: 47,810,529), RBPJ (chr6: 45,854,269), NKX2-1 (chr21: 46,713,013) and SOX5 (chr5: 86,055,295). Except for SOX5, they present similar expression profiles, falling in the upper cluster of Fig 3B. Importantly, those five genes were selected by different criteria: while NANOG and SOX5 were selected due to high expression in sperm cells, RBPJ and NKX2-1 were selected based on high connectivity, and HMGA2 was based on pleiotropy. In this sense, Fig 3C illustrates how the three selection criteria put forward different genes. Using this 3-way schema we were able to identify biologically relevant processes that could have been missed if using only one method had been used.

Stem cell differentiation is the process by which a cell is changed to a more specialized cell type. In the context of male fertility, spermatogonial stem cells go through a series of differentiation steps to become spermatozoa [39]. NANOG, a homeobox protein mostly associated with pluripotent cells, is also a key player in sperm cell differentiation of different mammalians [40]. It is not surprising then that it appears in the top 10 highly expressed genes in bovine sperm cells within our dataset. One positively regulated direct target of NANOG, at least in embryonic stem cells [41], is also within our key 29 TF. The Estrogen Related Receptor Beta (ESRRB) was shown to replace the NANOG function in pluripotent cells [41, 42] and our dataset demonstrates its regulatory potential by being the fifth most connected TF in our network. HMGA2 is part of the family of high mobility group proteins and is characteristic of rapidly dividing cells such as embryonic tissue and tumours [43]. It has also been shown to play an important role in male fertility, as the impairment of its function in mice was shown to block spermatogenesis [44]. Another gene controlling sperm cell proliferation and differentiation is RBPJ (Recombination Signal Binding Protein For Immunoglobulin Kappa J Region) which seems to be required for the proper regulation of the testis stem cell microenvironment [45]. SOX5 is one of the SRY-related box TF involved in steroidogenesis, spermatogenesis and sperm hyperactivation [46, 47]. While is not our intent to discuss all genes associated with relevant SNPs in this work, the relevance of several of these genes in the context of male fertility is evident.

The observed genomic correlations between the same trait in different breeds imply similarities in the genetic background across breeds. However, this was not observed across most traits. We confirmed that male fertility-related traits GEBV can be estimated with useful accuracy in a multi-breed scenario and that higher accuracies are obtained when the target breed is included in the reference population. Finally, functional variants/genes indicated through a 3-ways prioritization strategy are proposed as markers to assist in selection for male fertility traits. This multi-breed approach could be further developed in the future, aiming at a broader adoption of the technology by the industry.

## Materials and methods

### Animal care and use committee assessment

The total cattle population combined two research-based and four stud herds. The JM Rendel Laboratory Animal Experimentation Ethics Committee (CSIRO, Queensland, Australia) evaluated and approved the handling and sampling protocols of the two research-based herds (TBC107 and RH225-06, 1999–2006 and 2006–2010). For the four stud herds, historical DNA samples were accessed directly from a parent verification laboratory after receiving the consent of the bull owner. These samples were collected by bull breeders and sent to their service provider laboratory to confirm the parentage of their calves. No sample was collected, or cattle were handled for this project. In consultation with our local Animal Care and Use Committee, accessing archived historical samples was considered to fall outside the scope of the committee’s approval process.

### Animals and phenotypes

A total of 6,063 bulls from six breeds with measures for 10 phenotype traits were sourced from two research populations and four stud herds (S1 Table in S1 File). The two research populations comprised 1,051 Brahman (BRM) and 1,819 Tropical Composite (TRC) bulls and were part of a larger population established by the Cooperative Research Centre for Beef Genetic Technologies (Beef CRC) to understand the genetic links between adaptability and components of herd profitability in northern Australia [23, 48]. The four stud herds provided historical phenotypes and access to DNA (or hair) samples stored at a parent verification laboratory. The breeds represented by these four herds were Santa Gertrudis (SGT; N = 929 bulls), Droughtmaster (DMT; N = 760), Ultra Black (UBK; N = 844) and Belmont Tropical Composite (BTC; N = 660).

The 10 phenotypes included four measured on the bull and six measured on the bull’s semen (S2 Table in S1 File). Phenotypes measured on the bull included: body weight (WT, kg), body condition score (COND, score 1 to 5), scrotal circumference (SC, cm) measured with a standard measuring tape and sheath score (SHEATH, score 1 to 5). Semen samples were collected by an experienced technician using electroejaculation. The phenotypes measured on the bull’s semen included: the crush-side measurements of density (DENS, visual score 1 to 5), mass activity (MASS, visual score 1 to 5), progressive sperm motility (MOT, %), and the lab-based measurements of the percent normal sperm (PNS, %), percent sperm with proximal droplets (PD, %) and percent midpiece abnormalities (MP, %). A single observation was taken for each trait on each bull. For the research populations (BRM and TRC), SC was measured at yearling, while the other parameters, were measured at the first time the bull produced a semen sample. For all other populations, the traits were recorded as part of their routine preparation of the animals for sale.

The age at which the phenotypes were observed varied across the breeds; for BRM and TRC cattle, the age at SC was around 360 d, and for Sheath and PNS around 700 d. For SGT and DMT all phenotypes were observed at around 600 d of age, while for UBK and BTC at around 440 d and 390 d of age, respectively.

### Genotypes and genomic relationships

Most animals were genotyped using a commercial SNP chip with around ~50,000 markers. Genotypes were imputed up to high density (~700K SNP) using a reference population that combined Beef CRC and industry cattle genotyped on the higher density platform, mapped onto the ARS_UCD 1.2 cattle genome assembly [49]. Genotypes were first phased using Eagle [50] and then imputed using Minimac3 (autosomes) or Minimac4 (BTAX) [51]. SNP with imputation r^2^ > 0.8 were kept for further analyses. In total, 680,758 SNP were kept including 24,058 mapped on the X chromosome. We ascertained genomic structures based on principal components analyses (PCA) using PLINK1.9 [52] and computed genomic relationships employing within- and across-breed genomic relationship matrices (GRM) using Method 1 of VanRaden [53].

### Estimation of genetic parameters and genomic predictions

The phenotypes were adjusted before the genomic analyses for non-genetic effects using SAS software, Version 9.4 for PC. Copyright © [2002–2012] SAS Institute Inc. SAS and all other SAS Institute Inc. product or service names are registered trademarks or trademarks of SAS Institute Inc., Cary, NC, USA. The model for adjustment was as follows: y = Xβ + ε_1_ where y is the vector of values from a given phenotype, X is an incidence matrix relating the phenotypes with the fixed effects in β, which included the effects of population, year of birth and cohort, and the covariates of age and the first two principal components, and ε_1_ is the vector of random error assumed to be independent and normally distributed. The adjusted phenotypes (y*) were analyzed using a fully random model with polygenic (***u***) and residual effects to obtain estimates of genetic parameters and predicted genomic-estimated breeding values (GEBV) using the multi-variate genomic-relatedness-based restricted maximum likelihood (GREML) approach as implemented in Qxpak5 [54]. To estimate the genetic correlation between pairs of traits for the combined dataset with six breeds, a total of 45 bi-variate GREML analyses were performed for all pair-wise phenotypes (i.e., each using a GRM of dimension 6,063). In addition, following analytical approaches described in [21], the genomic correlation for a given phenotype in two breeds was estimated by treating each phenotype as a different trait in each breed pair. As a result, we performed a total of 150 bi-variates GREML analyses (i.e., from 15 pairs across the 6 breeds times the 10 phenotypes) each with a GRM of dimension equal to the number of animals in the breed pair.

### Quality assessment of genomic predictions

Traditional (or correlation-based) [55] and Method LR [22] approaches were used to estimate accuracy of GEBV. Using the second approach, bias and dispersion of GEBV were also estimated. Genomic estimates calculated using the whole dataset were defined as GEBV_Whole_ (or ${\hat{u}}_{w}$) and uni-variate models for cross-validation GEBV_Partial_ (or ${\hat{u}}_{p}$).

The following four metrics were employed:

1. Correlation-based Accuracy (ACC_R_): In the context of cross-validation, the accuracy of a GEBV is traditionally computed from the Pearson correlation between a GEBV and the adjusted phenotype (y*; phenotype *y* adjusted for fixed effects) for individuals in the validation population, and divided by the square root of heritability (*h*^*2*^):
$${ACC}_{R}=\frac{r({\hat{u}}_{p},y^{*})}{\sqrt{h^{2}}}$$
2. Method LR Accuracy (ACC_LR_): For individuals in the validation population, Method LR accuracy was computed as follows:
$${ACC}_{LR}=\sqrt{\frac{cov({\hat{u}}_{w},{\hat{u}}_{p})}{(1+\bar{F}-2\bar{f})\sigma_{g,\infty }^{2}}}$$
  Where $\bar{F}$ is the average inbreeding coefficient, $2\bar{f}$ is the average relationship between individuals, and $\sigma_{g,\infty }^{2}$ is the genetic variance at equilibrium in a population under selection. Assuming the individuals in the validation population are not under selection, $\sigma_{g,\infty }^{2}$ can be estimated by the additive genetic variance estimated from the partial dataset.
3. Method LR Bias (Bias_LR_): Difference between the average GEBV of individuals in the validation population using the partial data minus that using the whole data:
$${Bias}_{LR}=\bar{{\hat{u}}_{p}}-\bar{{\hat{u}}_{w}}$$
  In the absence of bias, the expected value of Bias_LR_ is zero.
4. Method LR Dispersion (Disp_LR_): For individuals in the validation population, dispersion was measured from the slope of the regression of ${\hat{u}}_{w}$ on ${\hat{u}}_{p}$:
$${Disp}_{LR}=1-\frac{cov({\hat{u}}_{w},{\hat{u}}_{p})}{var({\hat{u}}_{p})}$$
  In the absence of bias, the expected value of Disp_LR_ is 0. Values less than 0 indicate under-dispersion (or deflation) of ${\hat{u}}_{p}$ into ${\hat{u}}_{w}$ as phenotypes become available. Values greater than 1 indicate over-dispersion (or inflation) of ${\hat{u}}_{p}$ into ${\hat{u}}_{w}$.

### SNP effects, Association Weight Matrix (AWM), pleiotropy and gene co-association networks

We used the AWM methodology [56, 57] to identify and select the SNP to define the input data to construct a co-association network inferred using the Partial Correlation and Information Theory (PCIT) algorithm [58]. In brief, the AWM is built by generating normalized SNP effects across all ten traits, associating SNP to genes based on distance within the genome and selecting SNP/genes based on their significance to the key trait (PNS) or pleiotropic effect.

First, for each trait, SNP effects were estimated using derivations from [59, 60] as follows:

$$\hat{s}=\lambda M^{T}G^{-1}{\hat{u}}_{w}$$

In which $\hat{s}$ is the vector of estimated SNP effects of dimension 680,758 for as many SNP included in the analyses, *λ* is the ratio of SNP variance to genetic variance and assumed 0.85 throughout, **M** is the matrix of genotypes centred for observed allele frequencies with dimension equal to the number of animals (6,063) by number of SNP (680,758), **G** is the GRM computing using Method 1 of [53] across all animals and SNP, and ${\hat{u}}_{w}$ is as defined earlier for the trait of interest. SNP effects were z-score standardized and p-values for the association of an SNP to the trait obtained from an inversed normal distribution (S1–S3 Data). Second, SNP were mapped to genes. We used linkage disequilibrium (LD) findings from [61] where at short distances between markers (< 10 kb), taurine breeds showed higher LD (r^2^  =  0.45) than their indicine (r^2^  =  0.25) and composite (r^2^  =  0.32) counterparts. Therefore, we selected SNP located in the coding region or within 10 kb of an annotated gene based on the ARS_UCD1.2 bovine genome assembly [49]. Third, SNP (renamed by their linked gene) were included in the AWM if found to be either associated (p-value < 0.01) with the key phenotype (i.e. PNS) or pleiotropic. To capture pleiotropic SNP, we computed the average number of phenotypes to which the PNS-associated SNP were associated, namely *N*_PNS_, and selected the remaining SNP-Genes associated (p-value < 0.01) to more than *N*_PNS_ phenotypes. To further characterize the pleiotropic potential of SNP we computed the multi-trait *χ*^2^ statistic for the *i*-th SNP following derivations from [62]:

$$\chi_{i}^{T}={\hat{s}}_{i}^{T}V^{-1}{\hat{s}}_{i}$$

Where ${\hat{s}}_{i}$ is a 10 (number of traits) × 1 vector of z-score standardized effect of the *i-*th SNP and **V**^−1^ is the inverse of a 10 × 10 correlation matrix calculated overall estimated SNP effects. The *χ*^2^ value of each SNP was assessed for significance based on a *χ*^2^ distribution with 10 degrees of freedom to test against the null hypothesis that the SNP had no significant effect on any of the 10 traits. The final AWM contained as many rows as SNP (assigned to genes) and as many columns as traits, in this case ten traits.

To build the co-association network, we used the AWM as input, and identified significant gene-gene interactions using the PCIT algorithm [58] which calculates pairwise correlations between loci while accounting for the influence of a third locus. Unlike likelihood-based approaches, which invoke a parametric distribution (e.g., normal) assumed to hold under the null hypothesis and then a nominal p-value (e.g., 5%) used to ascertain significance, PCIT is an information theoretic approach. Its threshold is an informative metric; in this case, the partial correlation after exploring all trios in judging the significance of a given correlation, which might then become a connection when inferring a network. It thereby tests all possible 3-way combinations in a dataset and only keeps correlations between loci if they are significant and independent of another locus, whereas no hard threshold is set for the correlation strength. The significance threshold for each combination of loci depends on the average ratio of partial to direct correlations. Gene interactions were predicted using correlation analysis of the SNP effects across pairwise rows of the AWM. Hence, the AWM-predicted gene interactions are based on significant co-association between SNP. In the network, every node represents a gene (or SNP), whereas every edge connecting two nodes represents a significant gene-gene interaction (based on SNP-SNP co-association). Finally, the Cytoscape software [63] was used to visualize the gene network and the CentiScaPe plugin [64] was used to calculate specific node centrality values and network topology parameters. Functional enrichment analysis was performed using GeneMANIA [65].

### Identification of potential functional variants

To flag functional variants a 3-way search for key genes was performed by ranking transcription factors based on network connectivity, pleiotropy score and sperm-specific expression. To do that, we accessed the Animal Transcription Factor Database (AnimalTFDB3.0; [66], on March 24, 2021) to download a set of 1,396 *Bos taurus* genes annotated as transcription factors and the Cattle Gene Atlas [67] to explore the sperm specificity of gene expression based on the sperm epigenomic study of Liu et al., [68]. Variants associated to genes ranked in the top 10 positions in at least one of the three criteria were considered potential functional variants.

## Supporting information

S1 File (PDF)

S1 Data (GZ)

S2 Data (GZ)

S3 Data (GZ)

## References

[1] Eisler MC, Lee MRF, Tarlton JF, Martin GB, Beddington J, Dungait JAJ, et al. Agriculture: Steps to sustainable livestock. Nature. 2014;507: 32–34. doi: 10.1038/507032a 24605375

[2] Godfray HCJ, Aveyard P, Garnett T, Hall JW, Key TJ, Lorimer J, et al. Meat consumption, health, and the environment. Science (80-). 2018;361. doi: 10.1126/science.aam5324 30026199

[3] Boe‐Hansen GB, Rêgo JPA, Satake N, Venus B, Sadowski P, Nouwens A, et al. Effects of increased scrotal temperature on semen quality and seminal plasma proteins in Brahman bulls. Mol Reprod Dev. 2020;87: 574–597. doi: 10.1002/mrd.23328 32083367

[4] Morrell JM. Heat stress and bull fertility. Theriogenology. 2020;153: 62–67. doi: 10.1016/j.theriogenology.2020.05.014 32442741

[5] Lunstra DD, Ford JJ, Echternkamp SE. Puberty in Beef Bulls: Hormone Concentrations, Growth, Testicular Development, Sperm Production and Sexual Aggressiveness in Bulls of Different Breeds1. J Anim Sci. 1978;46: 1054–1062. doi: 10.2527/jas1978.4641054x 566747

[6] Fair S, Lonergan P. Review: Understanding the causes of variation in reproductive wastage among bulls. Animal. 2018;12: s53–s62. doi: 10.1017/S1751731118000964 29779500

[7] Lunstra DD, Cundiff L V. Growth and pubertal development in Brahman-, Boran-, Tuli-, Belgian Blue-, Hereford- and Angus-sired F1 bulls. J Anim Sci. 2003;81: 1414. doi: 10.2527/2003.8161414x 12817488

[8] Lyons RE, Loan NT, Dierens L, Fortes MRS, Kelly M, McWilliam SS, et al. Evidence for positive selection of taurine genes within a QTL region on chromosome X associated with testicular size in Australian Brahman cattle. BMC Genet. 2014;15: 6. doi: 10.1186/1471-2156-15-6 24410912 PMC3893399

[9] Hiltpold M, Kadri NK, Janett F, Witschi U, Schmitz-Hsu F, Pausch H. Autosomal recessive loci contribute significantly to quantitative variation of male fertility in a dairy cattle population. BMC Genomics. 2021;22: 225. doi: 10.1186/s12864-021-07523-3 33784962 PMC8010996

[10] Corbet NJ, Burns BM, Johnston DJ, Wolcott ML, Corbet DH, Venus BK, et al. Male traits and herd reproductive capability in tropical beef cattle. 2. Genetic parameters of bull traits. Anim Prod Sci. 2013;53: 101. doi: 10.1071/AN12163

[11] Butler ML, Hartman AR, Bormann JM, Weaber RL, Grieger DM, Rolf MM. Genetic parameter estimation for beef bull semen attributes. J Anim Sci. 2021;99. doi: 10.1093/jas/skab013 33453111 PMC8210814

[12] Bó GA, Baruselli PS. Synchronization of ovulation and fixed-time artificial insemination in beef cattle. Animal. 2014;8: 144–150. doi: 10.1017/S1751731114000822 24844128

[13] Baruselli PS, Ferreira RM, Sá Filho MF, Bó GA. Review: Using artificial insemination v. natural service in beef herds. Animal. 2018;12: s45–s52. doi: 10.1017/S175173111800054X 29554986

[14] Zhang YD, Johnston DJ, Bolormaa S, Hawken RJ, Tier B. Genomic selection for female reproduction in Australian tropically adapted beef cattle. Anim Prod Sci. 2014;54: 16. doi: 10.1071/AN13016

[15] Hayes BJ, Corbet NJ, Allen JM, Laing AR, Fordyce G, Lyons R, et al. Towards multi-breed genomic evaluations for female fertility of tropical beef cattle1. J Anim Sci. 2019;97: 55–62. doi: 10.1093/jas/sky417 30371787 PMC6313145

[16] Fordyce G, Entwistle K, Norman S, Perry V, Gardiner B, Fordyce P. Standardising bull breeding soundness evaluations and reporting in Australia. Theriogenology. 2006;66: 1140–1148. doi: 10.1016/j.theriogenology.2006.03.009 16620941

[17] Burns BM, Corbet NJ, Corbet DH, Crisp JM, Venus BK, Johnston DJ, et al. Male traits and herd reproductive capability in tropical beef cattle. 1. Experimental design and animal measures. Anim Prod Sci. 2013;53: 87. doi: 10.1071/AN12162

[18] Berry DP, Eivers B, Dunne G, McParland S. Genetics of bull semen characteristics in a multi-breed cattle population. Theriogenology. 2019;123: 202–208. doi: 10.1016/j.theriogenology.2018.10.006 30317043

[19] Fortes MRSS Porto-Neto LR, Satake N Nguyen LT, Freitas AC Melo TP, et al. X chromosome variants are associated with male fertility traits in two bovine populations. Genet Sel Evol. 2020;52: 1–13. doi: 10.1186/s12711-020-00563-5 32787790 PMC7425018

[20] Lund MS, Su G, Janss L, Guldbrandtsen B, Brøndum RF. Genomic evaluation of cattle in a multi-breed context. Livest Sci. 2014;166: 101–110. doi: 10.1016/j.livsci.2014.05.008

[21] Porto-Neto LR, Barendse W, Henshall JM, McWilliam SM, Lehnert SA, Reverter A. Genomic correlation: harnessing the benefit of combining two unrelated populations for genomic selection. Genet Sel Evol. 2015;47: 84. doi: 10.1186/s12711-015-0162-0 26525050 PMC4630892

[22] Legarra A, Reverter A. Semi-parametric estimates of population accuracy and bias of predictions of breeding values and future phenotypes using the LR method. Genet Sel Evol. 2018;50: 53. doi: 10.1186/s12711-018-0426-6 30400768 PMC6219059

[23] Porto-Neto LR, Reverter A, Prayaga KC, Chan EKF, Johnston DJ, Hawken RJ, et al. The Genetic Architecture of Climatic Adaptation of Tropical Cattle. Rueppell O, editor. PLoS One. 2014;9: e113284. doi: 10.1371/journal.pone.0113284 25419663 PMC4242650

[24] de Roos APW, Hayes BJ, Goddard ME. Reliability of Genomic Predictions Across Multiple Populations. Genetics. 2009;183: 1545–1553. doi: 10.1534/genetics.109.104935 19822733 PMC2787438

[25] Legarra A, Christensen OF, Vitezica ZG, Aguilar I, Misztal I. Ancestral Relationships Using Metafounders: Finite Ancestral Populations and Across Population Relationships. Genetics. 2015;200: 455–468. doi: 10.1534/genetics.115.177014 25873631 PMC4492372

[26] Junqueira VS, Lopes PS, Lourenco D, Silva FF e., Cardoso FF. Applying the Metafounders Approach for Genomic Evaluation in a Multibreed Beef Cattle Population. Front Genet. 2020;11. doi: 10.3389/fgene.2020.556399 33424914 PMC7793833

[27] Xiang R, Berg I van den, MacLeod IM, Hayes BJ, Prowse-Wilkins CP, Wang M, et al. Quantifying the contribution of sequence variants with regulatory and evolutionary significance to 34 bovine complex traits. Proc Natl Acad Sci. 2019;116: 19398–19408. doi: 10.1073/pnas.1904159116 31501319 PMC6765237

[28] Makinistoglu MP, Karsenty G. The class II histone deacetylase HDAC4 regulates cognitive, metabolic and endocrine functions through its expression in osteoblasts. Mol Metab. 2015;4: 64–69. doi: 10.1016/j.molmet.2014.10.004 25685691 PMC4314523

[29] Sujit KM, Sarkar S, Singh V, Pandey R, Agrawal NK, Trivedi S, et al. Genome-wide differential methylation analyses identifies methylation signatures of male infertility. Hum Reprod. 2018;33: 2256–2267. doi: 10.1093/humrep/dey319 30358834

[30] McDaneld TG, Kuehn LA, Thomas MG, Pollak EJ, Keele JW. Deletion on chromosome 5 associated with decreased reproductive efficiency in female cattle. J Anim Sci. 2014;92: 1378–1384. doi: 10.2527/jas.2013-6821 24492568

[31] Aguiar TS, Torrecilha RBP, Milanesi M, Utsunomiya ATH, Trigo BB, Tijjani A, et al. Association of Copy Number Variation at Intron 3 of HMGA2 With Navel Length in Bos indicus. Front Genet. 2018;9. doi: 10.3389/fgene.2018.00627 30581455 PMC6292862

[32] Kloth L, Gottlieb A, Helmke B, Wosniok W, Löning T, Burchardt K, et al. HMGA2 expression distinguishes between different types of postpubertal testicular germ cell tumour. J Pathol Clin Res. 2015;1: 239–251. doi: 10.1002/cjp2.26 27499908 PMC4939894

[33] Naval-Sánchez M, Porto-Neto LR, Cardoso DF, Hayes BJ, Daetwyler HD, Kijas J, et al. Selection signatures in tropical cattle are enriched for promoter and coding regions and reveal missense mutations in the damage response gene HELB. Genet Sel Evol. 2020;52: 27. doi: 10.1186/s12711-020-00546-6 32460767 PMC7251699

[34] Fortes MRS, Reverter A, Kelly M, McCulloch R, Lehnert SA. Genome-wide association study for inhibin, luteinizing hormone, insulin-like growth factor 1, testicular size and semen traits in bovine species. Andrology. 2013;1: 644–650. doi: 10.1111/j.2047-2927.2013.00101.x 23785023

[35] Xiang R, MacLeod IM, Daetwyler HD, de Jong G, O’Connor E, Schrooten C, et al. Genome-wide fine-mapping identifies pleiotropic and functional variants that predict many traits across global cattle populations. Nat Commun. 2021;12: 860. doi: 10.1038/s41467-021-21001-0 33558518 PMC7870883

[36] Alexandre PA, Naval-Sanchez M, Porto-Neto LR, Ferraz JBS, Reverter A, Fukumasu H. Systems biology reveals NR2F6 and TGFB1 as key regulators of feed efficiency in beef cattle. Front Genet. 2019;10: 1–16. doi: 10.3389/fgene.2019.00230 30967894 PMC6439317

[37] Mueller JL, Skaletsky H, Brown LG, Zaghlul S, Rock S, Graves T, et al. Independent specialization of the human and mouse X chromosomes for the male germ line. Nat Genet. 2013;45: 1083–1087. doi: 10.1038/ng.2705 23872635 PMC3758364

[38] Chen X, Wang Y, Zhu H, Hao H, Zhao X, Qin T, et al. Comparative transcript profiling of gene expression of fresh and frozen–thawed bull sperm. Theriogenology. 2015;83: 504–511. doi: 10.1016/j.theriogenology.2014.10.015 25459024

[39] Phillips BT, Gassei K, Orwig KE. Spermatogonial stem cell regulation and spermatogenesis. Philos Trans R Soc B Biol Sci. 2010;365: 1663–1678. doi: 10.1098/rstb.2010.0026 20403877 PMC2871929

[40] Kuijk EW, de Gier J, Chuva de Sousa Lopes SM, Chambers I, van Pelt AMM, Colenbrander B, et al. A Distinct Expression Pattern in Mammalian Testes Indicates a Conserved Role for NANOG in Spermatogenesis. Najbauer J, editor. PLoS One. 2010;5: e10987. doi: 10.1371/journal.pone.0010987 20539761 PMC2881870

[41] Zhang M, Leitch HG, Tang WWC, Festuccia N, Hall-Ponsele E, Nichols J, et al. Esrrb Complementation Rescues Development of Nanog-Null Germ Cells. Cell Rep. 2018;22: 332–339. doi: 10.1016/j.celrep.2017.12.060 29320730 PMC5775501

[42] Festuccia N, Osorno R, Halbritter F, Karwacki-Neisius V, Navarro P, Colby D, et al. Esrrb Is a Direct Nanog Target Gene that Can Substitute for Nanog Function in Pluripotent Cells. Cell Stem Cell. 2012;11: 477–490. doi: 10.1016/j.stem.2012.08.002 23040477 PMC3473361

[43] Fusco A, Fedele M. Roles of HMGA proteins in cancer. Nat Rev Cancer. 2007;7: 899–910. doi: 10.1038/nrc2271 18004397

[44] Chieffi P, Battista S, Barchi M, Di Agostino S, Pierantoni GM, Fedele M, et al. HMGA1 and HMGA2 protein expression in mouse spermatogenesis. Oncogene. 2002;21: 3644–3650. doi: 10.1038/sj.onc.1205501 12032866

[45] Garcia TX, Farmaha JK, Kow S, Hofmann M-C. RBPJ in mouse Sertoli cells is required for proper regulation of the testis stem cell niche. Development. 2014;141: 4468–4478. doi: 10.1242/dev.113969 25406395 PMC4302926

[46] Daigle M, Roumaud P, Martin LJ. Expressions of Sox9, Sox5, and Sox13 transcription factors in mice testis during postnatal development. Mol Cell Biochem. 2015;407: 209–221. doi: 10.1007/s11010-015-2470-7 26045173

[47] Mata-Rocha M, Hernández-Sánchez J, Guarneros G, de la Chesnaye E, Sánchez-Tusié AA, Treviño CL, et al. The transcription factors Sox5 and Sox9 regulate Catsper1 gene expression. FEBS Lett. 2014;588: 3352–3360. doi: 10.1016/j.febslet.2014.07.024 25101494

[48] Barwick SA, Johnston DJ, Burrow HM, Holroyd RG, Fordyce G, Wolcott ML, et al. Genetics of heifer performance in “wet” and “dry” seasons and their relationships with steer performance in two tropical beef genotypes. Anim Prod Sci. 2009;49: 367. doi: 10.1071/EA08273

[49] Rosen BD, Bickhart DM, Schnabel RD, Koren S, Elsik CG, Tseng E, et al. De novo assembly of the cattle reference genome with single-molecule sequencing. Gigascience. 2020;9. doi: 10.1093/gigascience/giaa021 32191811 PMC7081964

[50] Loh P-R, Danecek P, Palamara PF, Fuchsberger C, A Reshef Y, K Finucane H, et al. Reference-based phasing using the Haplotype Reference Consortium panel. Nat Genet. 2016;48: 1443–1448. doi: 10.1038/ng.3679 27694958 PMC5096458

[51] Das S, Forer L, Schönherr S, Sidore C, Locke AE, Kwong A, et al. Next-generation genotype imputation service and methods. Nat Genet. 2016;48: 1284–1287. doi: 10.1038/ng.3656 27571263 PMC5157836

[52] Chang CC, Chow CC, Tellier LCAM, Vattikuti S, Purcell SM, Lee JJ. Second-generation PLINK: rising to the challenge of larger and richer datasets. Gigascience. 2015;4: 7. doi: 10.1186/s13742-015-0047-8 25722852 PMC4342193

[53] VanRaden PM. Efficient methods to compute genomic predictions. J Dairy Sci. 2008;91: 4414–23. doi: 10.3168/jds.2007-0980 18946147

[54] Pérez-Enciso M, Misztal I. Qxpak.5: Old mixed model solutions for new genomics problems. BMC Bioinformatics. 2011;12: 202. doi: 10.1186/1471-2105-12-202 21612630 PMC3123239

[55] Bolormaa S, Pryce JE, Kemper K, Savin K, Hayes BJ, Barendse W, et al. Accuracy of prediction of genomic breeding values for residual feed intake and carcass and meat quality traits in Bos taurus, Bos indicus, and composite beef cattle1. J Anim Sci. 2013;91: 3088–3104. doi: 10.2527/jas.2012-5827 23658330

[56] Fortes MRS, Reverter A, Zhang Y, Collis E, Nagaraj SH, Jonsson NN, et al. Association weight matrix for the genetic dissection of puberty in beef cattle. Proc Natl Acad Sci. 2010;107: 13642–13647. doi: 10.1073/pnas.1002044107 20643938 PMC2922254

[57] Reverter A, Fortes MRS. Association Weight Matrix: A Network-Based Approach Towards Functional Genome-Wide Association Studies. Genome-wide association studies and genomic prediction. New York, NY: Humana Press; 2013. pp. 437–447. doi: 10.1007/978-1-62703-447-0_20 23756904

[58] Reverter A, Chan EKF. Combining partial correlation and an information theory approach to the reversed engineering of gene co-expression networks. Bioinformatics. 2008;24: 2491–2497. doi: 10.1093/bioinformatics/btn482 18784117

[59] Strandén I, Garrick DJ. Technical note: Derivation of equivalent computing algorithms for genomic predictions and reliabilities of animal merit. J Dairy Sci. 2009;92: 2971–2975. doi: 10.3168/jds.2008-1929 19448030

[60] WANG H, MISZTAL I, AGUILAR I, LEGARRA A, MUIR WM. Genome-wide association mapping including phenotypes from relatives without genotypes. Genet Res (Camb). 2012;94: 73–83. doi: 10.1017/S0016672312000274 22624567

[61] Porto-Neto LR, Kijas JW, Reverter A. The extent of linkage disequilibrium in beef cattle breeds using high-density SNP genotypes. Genet Sel Evol. 2014;46: 22. doi: 10.1186/1297-9686-46-22 24661366 PMC4021229

[62] Bolormaa S, Pryce JE, Reverter A, Zhang Y, Barendse W, Kemper K, et al. A Multi-Trait, Meta-analysis for Detecting Pleiotropic Polymorphisms for Stature, Fatness and Reproduction in Beef Cattle. Flint J, editor. PLoS Genet. 2014;10: e1004198. doi: 10.1371/journal.pgen.1004198 24675618 PMC3967938

[63] Shannon P, Markiel A, Ozier O, Baliga NS, Wang JT, Ramage D, et al. Cytoscape: a software environment for integrated models of biomolecular interaction networks. Genome Res. 2003;13: 2498–2504. doi: 10.1101/gr.1239303 14597658 PMC403769

[64] Scardoni G, Petterlini M, Laudanna C. Analyzing biological network parameters with CentiScaPe. Bioinformatics. 2009;25: 2857–2859. doi: 10.1093/bioinformatics/btp517 19729372 PMC2781755

[65] Warde-Farley D, Donaldson SL, Comes O, Zuberi K, Badrawi R, Chao P, et al. The GeneMANIA prediction server: Biological network integration for gene prioritization and predicting gene function. Nucleic Acids Res. 2010;38: 214–220. doi: 10.1093/nar/gkq537 20576703 PMC2896186

[66] Hu H, Miao Y-R, Jia L-H, Yu Q-Y, Zhang Q, Guo A-Y. AnimalTFDB 3.0: a comprehensive resource for annotation and prediction of animal transcription factors. Nucleic Acids Res. 2019;47: D33–D38. doi: 10.1093/nar/gky822 30204897 PMC6323978

[67] Fang L, Cai W, Liu S, Canela-Xandri O, Gao Y, Jiang J, et al. Comprehensive analyses of 723 transcriptomes enhance genetic and biological interpretations for complex traits in cattle. Genome Res. 2020;30: 790–801. doi: 10.1101/gr.250704.119 32424068 PMC7263193

[68] Liu S, Chen S, Cai W, Yin H, Liu A, Li Y, et al. Divergence Analyses of Sperm DNA Methylomes between Monozygotic Twin AI Bulls. Epigenomes. 2019;3: 21. doi: 10.3390/epigenomes3040021 34968253 PMC8594723

