# Supplementary material for: Multi-breed genomic predictions and functional variants for fertility of tropical bulls
Source: PLoS One. 2023 Jan 26;18(1):e0279398. doi: 10.1371/journal.pone.0279398 (PMC9879470; doi:10.1371/journal.pone.0279398)
Supplement: S1 File — (PDF) [file pone.0279398.s001.pdf]

Supplementary Material

Multi-breed genomic predictions and functional variants for fertility of tropical bulls

Laercio R. Porto-Neto[1,*], Pamela A. Alexandre[1], Nicholas J. Hudson[2], John Bertram[3], Sean M. McWilliam[1], Andre W.L. Tan[4], Marina R.S. Fortes[4], Michael R. McGowan[5], Ben J. Hayes[6] and Antonio Reverter[1]

[1] CSIRO Agriculture & Food, St Lucia, QLD 4067 Australia.

[2] The University of Queensland, School of Animal Studies, Gatton, QLD 4343 Australia.

[3] Agriculture Consultant, Livestock management and breeding, Toowoomba QLD 4350 Australia.

[4] The University of Queensland, School of Chemistry and Molecular Bioscience, St Lucia, QLD 4072 Australia.

[5] The University of Queensland, School of Veterinary Sciences, Gatton, QLD 4343 Australia.

[6] The University of Queensland, Queensland Alliance for Agriculture and Food Innovation, St Lucia, QLD 4072 Australia.

[*] corresponding author: Laercio.Portoneto@csiro.au

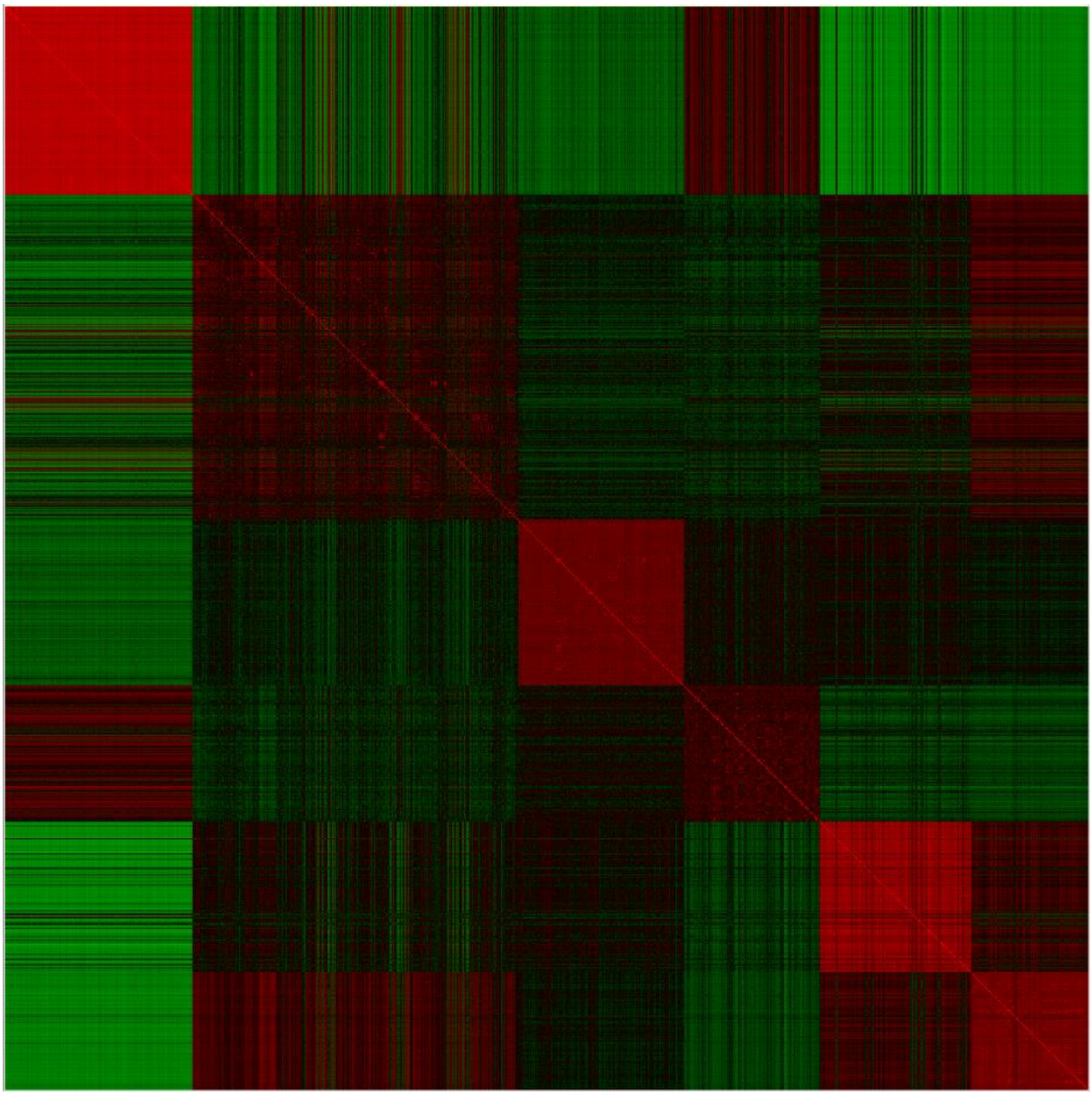

S1 Fig. Heatmap of the genomic relationship matrix (GRM). Genomic relationships estimated across the 6,063 bulls clearly shown the presence of six clusters along the diagonal corresponding to the six breeds represented.

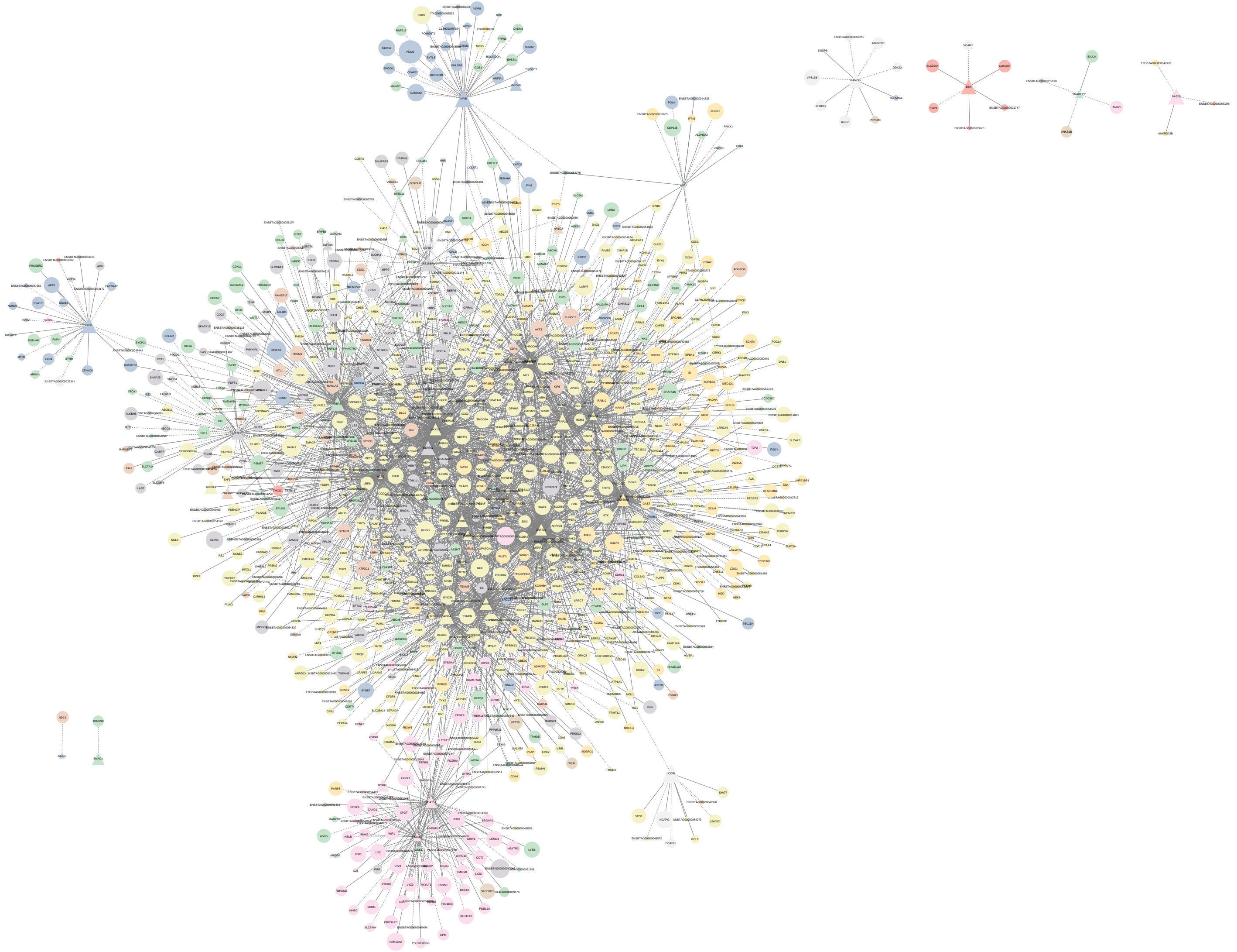

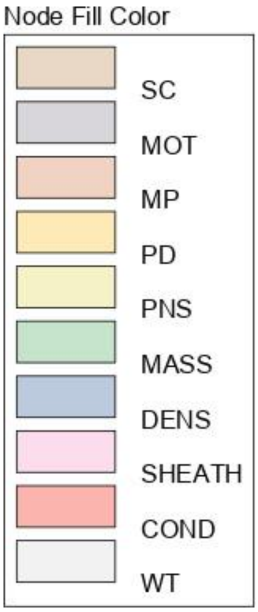

S2 Fig. Full network of genes highlighted through functionality analysis. Colors correspond to the most associated trait (SC = scrotal circumference; MOT = motility; MD = midpiece abnormalities PD = proximal cytoplasmic droplets; PNS = percent normal sperm; MASS = mass; DENS = density; SHEATH= sheath score; COND = condition score; and WT = body weight).
(Zoon in at the network to read the details).

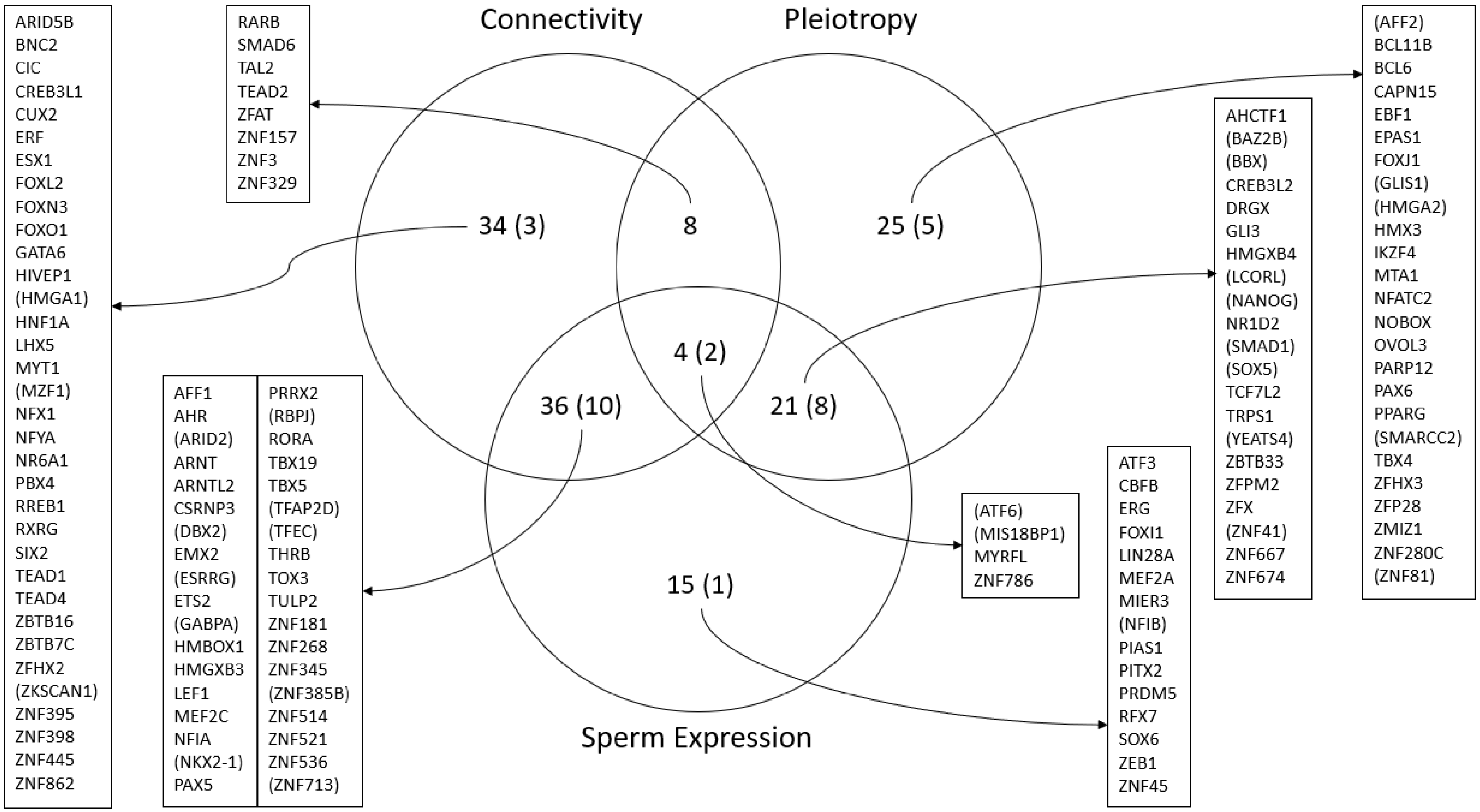

S3 Fig. Transcription factors (TF) selected for being above average according to connectivity, pleiotropy, and sperm expression. The 29 top10 TF for the three selection criteria are represented in parenthesis.

S1 Table. Number of records by breed and trait.

Breed	Total	Trait									
		WT	COND	SC	SHEATH	DENS	MASS	MOT	PNS	PD	MP
BRM	1,051	1,051	1,051	1,051	1,051	1,051	1,051	1,051	1,051	1,051	1,051
TRC	1,819	1,819	1,819	1,819	1,819	1,818	1,817	1,818	1,819	1,819	1,819
SGT	929	929	928	918	928	901	901	901	895	895	895
DMT	760	750	617	601	760	581	581	581	710	709	710
UBK	844	454	842	837	842	785	785	785	783	781	781
BTC	660	655	660	653	660	453	453	453	450	450	450
Total	6,063	5,658	5,917	5,879	6,060	5,589	5,588	5,589	5,708	5,705	5,706

Breeds include Brahman (BRM), Tropical Composite (TRC), Santa Gertrudis (SGT), Droughtmaster (DMT), Ultra-black (UBK) and Belmont Tropical Composite (BTC). Traits include WT – weight (Kg), COND – body condition score (1-5), SC – scrotal circumference (cm), SHEATH – Sheath score (1-6), DENS – Density of ejaculate (1-5), MASS – mass movement of sperm in ejaculate (1-5), MOT – sperm progressive motility (%), PNS – percentage of normal sperm (%), PD – proximal cytoplasmic droplets (%), MP – middle piece abnormalities (%).

S2 Table. Average (SD) measurement by breed and trait.

Breed	WT	COND	SC	SHEATH	DENS	MASS	MOT	PNS	PD	MP
BRM	359.05 (42.9)	3.09 (0.37)	27.92 (2.80)	5.88 (1.11)	2.64 (0.95)	2.09 (1.07)	60.22 (23.66)	51.57 (30.24)	24.50 (27.16)	13.40 (11.25)
TRC	328.50 (58.7)	2.81 (0.44)	28.89 (2.89)	3.13 (1.77)	2.80 (0.94)	2.35 (1.07)	64.71 (22.11)	57.63 (27.23)	14.51 (19.79)	14.34 (12.47)
SGT	507.28 (79.2)	3.03 (0.28)	34.46 (3.10)	2.94 (0.78)	2.67 (0.94)	2.25 (0.93)	62.83 (21.50)	73.35 (21.43)	6.88 (12.50)	7.85 (8.28)
DMT	459.53 (58.0)	3.05 (0.29)	33.65 (3.16)	3.14 (0.68)	2.39 (0.91)	2.15 (0.93)	69.59 (20.85)	65.69 (25.16)	9.88 (16.36)	8.93 (9.67)
UBK	439.16 (65.7)	3.08 (0.23)	33.80 (3.37)	1.78 (0.80)	2.58 (0.78)	2.56 (0.88)	73.58 (19.00)	67.66 (26.57)	10.20 (15.96)	7.83 (8.24)
BTC	283.68 (54.2)	2.83 (0.25)	27.27 (3.77)	1.64 (0.58)	2.14 (0.79)	2.03 (0.90)	66.04 (24.61)	56.14 (28.07)	13.80 (18.35)	13.66 (11.96)

Breeds include Brahman (BRM), Tropical Composite (TRC), Santa Gertrudis (SGT), Droughtmaster (DMT), Ultra-black (UBK) and Belmont Tropical Composite (BTC). Traits include WT – weight (Kg), COND – body condition score (1-5), SC – scrotal circumference (cm), SHEATH – Sheath score (1-6), DENS – Density of ejaculate (1-5), MASS – mass movement of sperm in ejaculate (1-5), MOT – sperm progressive motility (%), PNS – percentage of normal sperm (%), PD – proximal cytoplasmic droplets (%), MP – middle piece abnormalities (%).

S3 Table. ANOVA P-values for the strength of the association of heritability (h^2), breed, phenotype and cross-validation split (Xval) on correlation-based accuracy (ACC_R) and method LR accuracy (ACC_{LR}), bias and dispersion (Disp.) of GEBV two validation schemes and percentage of variation (R^2) explained by the ANOVA model.

Effect	Validation Scheme #1				Validation Scheme #2			
	ACC_R	ACC_{LR}	Bias	Disp.	ACC_R	ACC_{LR}	Bias	Disp.
h^2	0.0281	0.0001	0.8765	0.0001	0.1231	0.0017	0.8799	0.0001
Breed	0.0012	0.0030	0.8423	0.0075	0.0001	0.0001	0.9742	0.0001
Phenotype	0.0001	0.0001	0.1543	0.0001	0.0001	0.0001	1.0000	0.0001
Xval	NA	NA	NA	NA	0.1010	0.9724	0.9828	0.0193
R^2, %	77.5	85.1	27.5	69.7	55.6	71.9	0.50	70.1

Validation Scheme #1: From a given validation breed, all measures set as missing in the reference population.
Validation Scheme #2: From a given validation breed, a random 20% of measures missing in the reference (and then averaged across the five 80/20 cross-validation splits).

S4 Table. Functional enrichment of 29 genes selected as top10 for connectivity, pleiotropy, and sperm expression.

Function	FDR	Genes in network	Genes in genome
chromatin remodeling	1.31E-06	8	92
transcription coactivator activity	2.41E-06	10	249
protein-DNA complex disassembly	2.41E-06	5	17
nucleosome disassembly	2.41E-06	5	17
chromatin disassembly	2.61E-06	5	18
chromatin assembly or disassembly	4.62E-06	7	83
nucleosome organization	6.34E-05	6	71
sequence-specific DNA binding RNA polymerase II transcription factor activity	7.94E-05	8	200
SWI/SNF complex	1.01E-04	4	15
RNA polymerase II transcription regulatory region sequence-specific DNA binding transcription factor activity involved in positive regulation of transcription	1.24E-04	5	42
BAF-type complex	1.84E-04	4	18
protein-DNA complex subunit organization	2.01E-04	6	94
RNA polymerase II core promoter proximal region sequence-specific DNA binding transcription factor activity involved in positive regulation of transcription	2.58E-03	4	35
npBAF complex	3.35E-03	3	11
nBAF complex	4.16E-03	3	12
regulatory region DNA binding	4.51E-03	7	268
regulatory region nucleic acid binding	4.51E-03	7	268
**stem cell differentiation**	**4.51E-03**	**6**	**171**
transcription regulatory region DNA binding	4.51E-03	7	267
RNA polymerase II core promoter proximal region sequence-specific DNA binding transcription factor activity	1.39E-02	4	59
SWI/SNF superfamily-type complex	1.72E-02	4	63
regulation of organ morphogenesis	1.86E-02	4	65
**regulation of stem cell differentiation**	**2.39E-02**	**4**	**70**
epithelial to mesenchymal transition	2.43E-02	4	71
RNA polymerase II core promoter proximal region sequence-specific DNA binding transcription factor activity involved in negative regulation of transcription	2.55E-02	3	25
RNA polymerase II transcription regulatory region sequence-specific DNA binding transcription factor activity involved in negative regulation of transcription	3.11E-02	3	27
protein complex disassembly	3.14E-02	5	156
BMP signaling pathway	3.17E-02	4	79
macromolecular complex disassembly	3.60E-02	5	163
artery morphogenesis	3.72E-02	3	30
artery development	4.81E-02	3	33
